# Crystal Structure of DmdD, a Crotonase Superfamily Enzyme That Catalyzes the Hydration and Hydrolysis of Methylthioacryloyl-CoA

**DOI:** 10.1371/journal.pone.0063870

**Published:** 2013-05-21

**Authors:** Dazhi Tan, Warren M. Crabb, William B. Whitman, Liang Tong

**Affiliations:** 1 Department of Biological Sciences, Columbia University, New York, New York, United States of America; 2 Department of Microbiology, University of Georgia, Athens, Georgia, United States of America; University of Melbourne, Australia

## Abstract

Dimethyl-sulphoniopropionate (DMSP) is produced in abundance by marine phytoplankton, and the catabolism of this compound is an important source of carbon and reduced sulfur for marine bacteria and other organisms. The enzyme DmdD catalyzes the last step in the methanethiol (MeSH) pathway of DMSP catabolism. DmdD is a member of the crotonase superfamily of enzymes, and it catalyzes both the hydration and the hydrolysis of methylthioacryloyl-CoA (MTA-CoA), converting it to acetaldehyde, CO_2_, MeSH, and CoA. We report here the crystal structure of *Ruegeria pomeroyi* DmdD free enzyme at 1.5 Å resolution and the structures of the E121A mutant in complex with MTA-CoA and 3-methylmercaptopropionate-CoA (MMPA-CoA) at 1.8 Å resolution. DmdD is a hexamer, composed of a dimer of trimers where the three monomers of each trimer are related by a crystallographic 3-fold axis. The overall structure of this hexamer is similar to those of canonical crotonases. However, the C-terminal loops of DmdD in one of the trimers assume a different conformation and contribute to CoA binding in the active site of a neighboring monomer of the trimer, while these loops in the second trimer are disordered. MTA-CoA is bound deep in the active site in the first trimer, but shows a 1.5 Å shift in its position in the second trimer. MMPA-CoA has a similar binding mode to MTA-CoA in the first trimer. MMPA-CoA cannot be hydrated and is only hydrolyzed slowly by DmdD. Replacement of the sulfur atom in MMPA-CoA with a methylene group abolishes hydrolysis, suggesting that the unique property of the substrate is a major determinant of the hydrolysis activity of DmdD.

## Introduction

**Figure 1:**
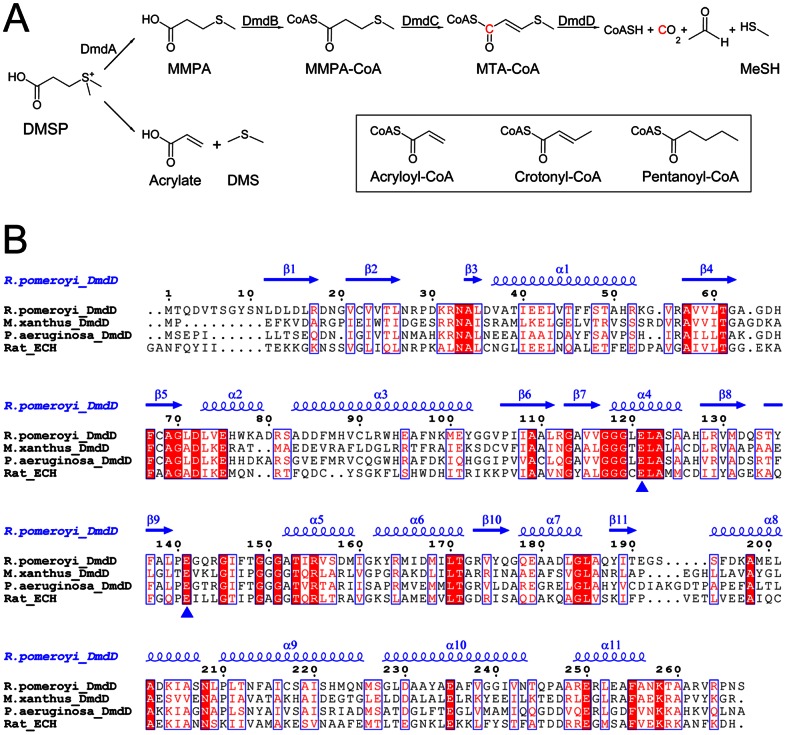
Sequence conservation of DmdD. (**A**). The MeSH and DMS catabolic pathways of DMSP. The enzymes in the MeSH parthway are indicated. The chemical structures of acryloyl-CoA, crotonyl-CoA and pentanoyl-CoA are shown in the inset. (**B**). Sequence alignment of *Ruegeria pomeroyi* DmdD, *Myxococcus xanthus* DmdD, *Pseudomonas aeruginosa* DmdD, and rat liver enoyl-CoA hydratase (ECH). The secondary structure elements in the structure of *R. pomeroyi* DmdD are labeled. The two catalytic Glu residues are indicated with the blue triangles. The residue numbers refer to *R. pomeroyi* DmdD.

Dimethyl-sulphoniopropionate (DMSP) is produced in abundant quantities by marine surface-water phytoplankton, including coccolithophores, dinoflagellates, and diatoms, as well as some plants common in salt marshes. In these organisms, the compound plays a role in osmoregulation, stress control, detoxification, and other functions [1], [2], [3], [4]. DMSP is an important source of carbon and reduced sulfur for marine bacteria. It is catabolized through two competing pathways, releasing either the climatically active gas dimethylsulphide (DMS) or the highly reactive volatile gas methanethiol (MeSH) (Fig. 1A). For the DMS pathway, a lyase catalyzes the cleavage of DMSP, producing DMS and acrylate (or occasionally 3-hydroxypropionate in some bacteria). For the MeSH pathway, DMSP is first demethylated to produce 3-methylmercaptopropionate (MMPA), which is then esterified to coenzyme A (CoA). MMPA-CoA is demethiolated through a set of reactions analogous to β-oxidation of fatty acids—a dehydrogenation reaction produces methylthioacryloyl-CoA (MTA-CoA) followed by a hydration reaction that ultimately converts MMPA to acetaldehyde, CO_2_, and MeSH (Fig. 1A).

The enzyme DmdD catalyzes the last step of the MeSH pathway, the hydration and hydrolysis of MTA-CoA (Fig. 1A) [2]. DmdD belongs to the crotonase superfamily of enzymes, homologous to the equivalent enzyme in the fatty acid β-oxidation pathway. For example, *Ruegeria pomeroyi* DmdD shares 32% amino acid sequence identity with rat liver enoyl-CoA hydratase (ECH), a canonical crotonase (Fig. 1B). Of special interest, the two acidic residues that are important for the catalysis of ECH (Glu144 and Glu164) are also conserved in DmdD (Glu121 and Glu141). Enzymes in the crotonase superfamily are mechanistically diverse and catalyze many different types of reactions on CoA esters [5], including hydration [6], hydrolysis [7], [8], [9], [10], isomerization [11], dehalogenation [12], decarboxylation [13], and others.

To understand the molecular basis for the unique catalytic activities of DmdD, we have determined the crystal structures of *R. pomeroyi* wild-type DmdD free enzyme and the E121A mutant in complex with MTA-CoA or MMPA-CoA at 1.5, 1.8, and 1.8 Å resolution, respectively. Our structures reveal conformational differences for the C-terminal loop of DmdD compared to canonical crotonases, which affect the organization of the active site. MTA-CoA and MMPA-CoA have similar binding modes in the active site. However, MMPA-CoA cannot be hydrated and is only hydrolyzed slowly by DmdD. Replacement of the sulfur atom in MMPA-CoA with a methylene group abolishes hydrolysis, suggesting that the unique property of the substrate is a major determinant of the hydrolysis activity of DmdD.

## Results and Discussion

### Structure of DmdD monomer

The crystal structure of wild-type *R. pomeroyi* DmdD free enzyme has been determined at 1.5 Å resolution by the molecular replacement method. To determine the structure of DmdD in complex with its substrate, we created the catalytically inactive E121A mutant (see below). Crystals of this mutant were soaked with MTA-CoA or MMPA-CoA, and the structures of the two complexes were determined at 1.8 Å resolution. The atomic models have excellent agreement with the X-ray diffraction data and the expected bond lengths, bond angles and other geometric parameters (Table 1). All the residues are located in the favored regions of the Ramachandran plot (data not shown).

**Table 1:** Summary of crystallographic information.

Structure	Wild-type Free enzyme	E121A mutant MTA-CoA complex	E121A mutant MMPA-CoA complex
Resolution range (Å)^1^	30-1.5 (1.55-1.5)	50-1.8 (1.86-1.8)	50-1.8 (1.86-1.8)
Number of observations	568,858	317,169	359,988
*R*_merge_ (%)	4.6 (21.9)	8.2 (31.5)	5.6 (45.7)
I/σI	31.7 (7.8)	21.6 (5.4)	32.6 (4.5)
Redundancy	6.7 (6.3)	6.3 (6.0)	7.3 (6.9)
Completeness (%)	96.1 (85.1)	98.3 (94.7)	96.3 (87.5)
*R* factor^2^ (%)	13.3 (13.2)	15.9 (26.8)	16.0 (23.5)
free *R* factor^2^ (%)	16.0 (17.2)	18.1 (30.1)	18.7 (24.6)
rms deviation in bond lengths (Å)	0.013	0.010	0.009
rms deviation in bond angles (°)	1.6	1.5	1.4
Number of protein atoms	3,737	3,908	3,865
Number of ligand atoms	0	108	99
Number of waters	588	517	441
PDB entry codes	4IZB	4IZC	4IZD

1 The numbers in parentheses are for the highest resolution shell.

**Figure 2:**
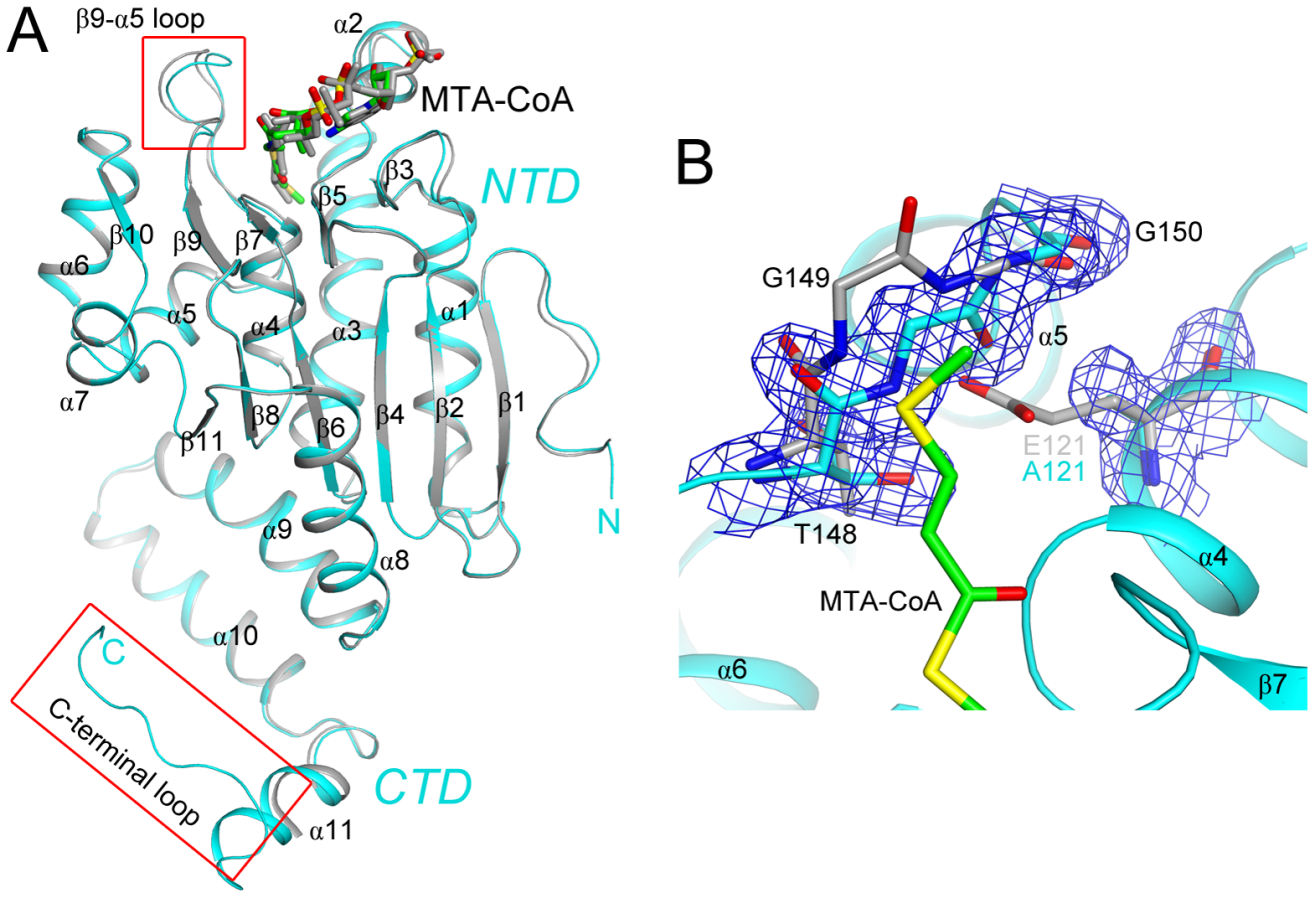
Structure of the DmdD monomer. (**A**). Overlay of the structures of the two DmdD (E121A mutant) molecules in complex with MTA-CoA in the asymmetric unit of the crystal. One molecule is colored in cyan, and the other in gray. The MTA-CoA molecules are shown in stick models. The red boxes highlight regions of structural differences between the two molecules. (**B**). Omit F_o_–F_c_ electron density map at 1.8 Å resolution, contoured at 3σ, for residues 121 and 148–150 in the structure of the E121A mutant of DmdD (in cyan) in complex with MTA-CoA (in green). The structure of these residues in wild-type DmdD is shown for comparison (in gray). The flip of the Gly149–Gly150 peptide bond is likely linked to the E121A mutation. All structure figures were produced with PyMOL (www.pymol.org).

The structure of the DmdD monomer contains two domains. The N-terminal domain (NTD) adopts the typical spiral crotonase fold (β-β-αsuperhelix, β1–β11 and α1–α8), which is organized around two roughly perpendicular β-sheets (Fig. 2A). The C-terminal domain (CTD) consists of three α helices (α9–α11) followed by a long loop at the extreme C-terminus of the protein. The CTD mediates the hexamerization of DmdD, and the long loop at the C-terminus also participates in the formation of the CoA binding site of a neighboring monomer of the hexamer (see below).

The overall conformations of the six DmdD monomers in the three structures are highly similar to each other, with rms distance of 0.2 to 0.5 Å between equivalent Cα atoms of any pair of the monomers. The two monomers in the free enzyme and the equivalent monomers in the MMPA-CoA and MTA-CoA complexes have rms distances of ∼0.2 Å for their Cα atoms.

There are also recognizable differences among the structures. For example, the long loop at the C-terminus of DmdD is observed only in one of the two monomers in the MTA-CoA complex (Fig. 2A), where it is involved in binding the substrate. A shorter segment of this loop is observed in the equivalent monomer in the MMPA-CoA complex. In contrast, this loop and part of helix α11 are disordered in the other monomers (Fig. 2A). In the active site region, there are large differences in the conformation of residues 139–146 (the β9-α5 loop) between the two monomers in the MTA-CoA or the MMPA-CoA complexes (Fig. 2A), which may be related to the positioning of the substrates (see below). Moreover, the Gly149–Gly150 peptide bond is flipped in the structures of the complexes as compared to that of the free, wild-type enzyme. This is likely due to the E121A mutation, as the peptide bond in the flipped position occupies the position of the Glu121 side chain in the wild-type enzyme structure (Fig. 2B). However, it is unlikely that this flipped position of the Gly149–Gly150 peptide bond has affected the binding mode of MTA-CoA or MMPA-CoA.

### The hexamer of DmdD

**Figure 3:**
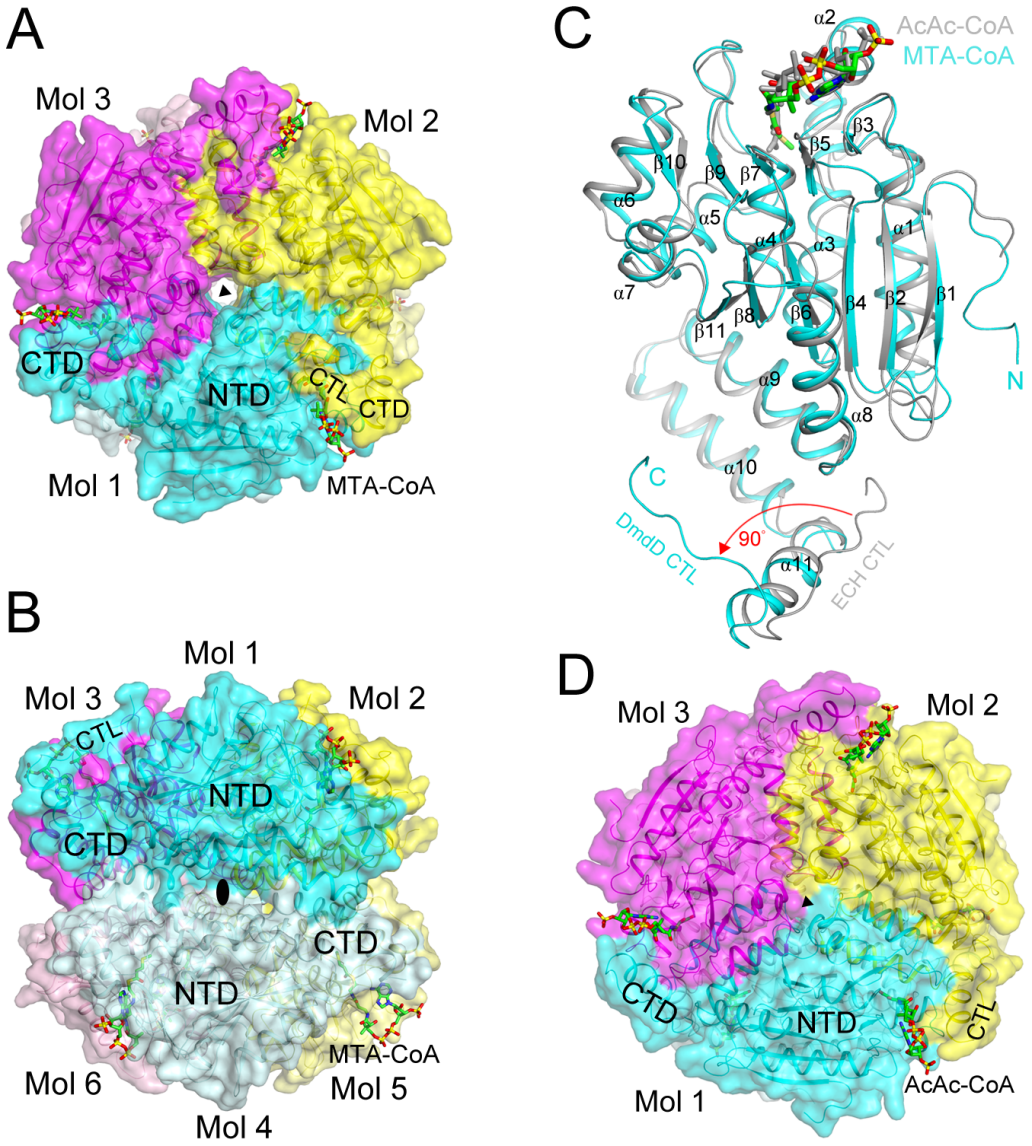
Structure of the DmdD hexamer. (**A**). Structure of the DmdD E121A mutant hexamer in complex with MTA-CoA, viewed down the three-fold symmetry axis (black triangle). The three molecules of the trimer are given different colors, and the MTA-CoA molecules are shown in stick models (in green). The N-terminal domain (NTD), C-terminal domain (CTD), and C-terminal loop (CTL) are labeled. (**B**). Structure of the DmdD hexamer, viewed from the side, down a two-fold symmetry axis (black oval). The six molecules of the hexamer are labeled. (**C**). Overlay of the structures of the DmdD E121A mutant monomer (in cyan) in complex with MTA-CoA (in green) and rat liver ECH monomer in complex with AcAc-CoA (in gray) [15]. A large difference in the conformation of the C-terminal loop (CTL) of the two structures is indicated in red. (**D**). Structure of the rat liver ECH hexamer in comple with AcAc-CoA, viewed down the three-fold symmetry axis (black triangle). The CoA binding region is more open to the solvent compared to that in DmdD (panel A).

Consistent with the gel-filtration data, DmdD is a hexamer in the crystal, which is also similar to the architecture of canonical crotonases. The hexamer is generated from the two monomers in the asymmetric unit by the crystallographic 3-fold symmetry axis (Fig. 3A), and can be regarded as two layers of DmdD trimers (Fig. 3B). The DmdD trimer is formed by intimate contacts between the CTD (α9–α11) and NTD (α2, α3, α6 and α7) of neighboring monomers in the same layer. Approximately 4,700 Å^2^ of the surface area of each monomer is buried at the trimer interface. In comparison, the hexamer interface is less extensive and involves predominantly the CTD of the six monomers (Fig. 3B), with each monomer of DmdD contributing 1,200 Å^2^ surface area to the hexamer interface.

The active site is located at the trimer interface, and the three active sites in each trimer are identical to each other due to the crystallographic symmetry (Fig. 3A). On the other hand, the active sites in the two layers of the hexamer show differences (Fig. 3B), both in the enzyme and in the binding modes of the substrates (see below).

### Structural comparison to canonical crotonases

The overall structure of DmdD monomer is similar to those of other crotonases. A search through the Protein Data Bank, with the program DaliLite [14], finds a large number of structural homologs, with *Z* scores above 20. A detailed comparison will be made here with the structure of rat liver enoyl-CoA hydratase (ECH), a canonical hexameric crotonase, in complex with the inhibitor acetoacetyl-CoA (AcAc-CoA) [15]. The rms distance between 214 equivalent Cα atoms of ECH and DmdD is 1.5 Å (Fig. 3C). The positions of most of the secondary structure elements (including the three helices in the CTD) are similar in the two structures. The overall organization of the hexamer of ECH is also similar to that of DmdD (Fig. 3D).

There is one striking structural difference, however, between DmdD and ECH. The C-terminal loop (CTL) in ECH is positioned nearly 90° away from that in DmdD (Fig. 3C). This loop has interactions with the phosphate groups of CoA in DmdD (Fig. 3A, see below). In comparison, the loop in ECH has few interactions with CoA, and as a result the phosphate groups are more exposed to the solvent in ECH (Fig. 3D).

### Two different binding modes of MTA-CoA

**Figure 4:**
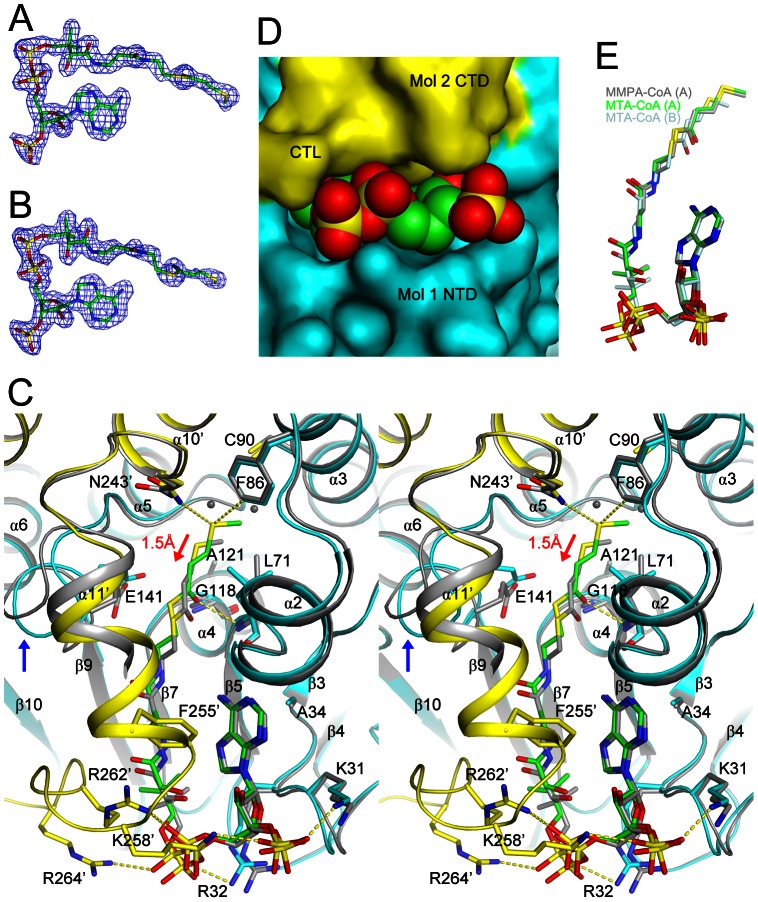
The active site of DmdD. (**A**). Omit F_o_–F_c_ electron density map at 1.8 Å resolution for MTA-CoA in binding mode A, contoured at 3σ. (**B**). Omit F_o_–F_c_ electron density map for MTA-CoA in binding mode B, contoured at 2.5σ. (**C**). Overlay of the two DmdD active sites in the crystal of the E121A mutant in complex with MTA-CoA (in stereo). For binding mode A, molecule 1 of DmdD is shown in cyan, molecule 3 in yellow, and MTA-CoA in green. The MTA-CoA molecule in binding mode B and the corresponding binding residues in DmdD are shown in gray. The red arrow indicates the shift in the position of MTA-CoA in binding mode B relative to binding mode A. The blue arrow indicates conformational differences for the β9-α5 loop (include Glu141) between the two DmdD molecules. Close neighbors of the sulfur atom in binding mode A are indicated with the dashed lines (yellow). (**D**). Solvent accessible surface of the active site region of DmdD, corresponding to binding mode A of MTA-CoA (in green). (**E**). Overlay of binding modes A (in green) and B (in light cyan) of MTA-CoA and binding mode A of MMPA-CoA (in gray).

As in other crotonases, CoA is located on top of the small β-sheet of the spiral crotonase fold (Fig. 2A). Good-quality electron density was observed for both MTA-CoA molecules in the crystal (Figs. 4A and 4B). However, there are differences in their binding modes in the two molecules of DmdD. In the first molecule (molecule 1 in Fig. 3A), the entire MTA-CoA molecule (Fig. 4A) is inserted into a deep active site pocket on the surface of DmdD (to be referred to as binding mode A), formed primarily by residues in one monomer (α2, α3, α10, β5-α2 loop, and β9-α5 loop) but also with contributions from the CTD (α10, α11 and the C-terminal loop) of a neighboring monomer of the trimer (Figs. 3A and 4C). The N1 and N6 atoms of the adenine base are recognized by hydrogen-bonds with the main-chain amide of Leu73 and main-chain carbonyl of Leu71, respectively. The adenine base is flanked by the side chain of Ala34 on one face and Phe255′ from the CTD of the neighboring monomer on the other face (with the prime indicating the neighboring monomer). The phosphate groups of CoA are recognized by ionic interactions with Lys31, Arg32, and His66. Moreover, the C-terminal loop of the neighboring monomer provides three additional side chains, Lys258′, Arg262′ and Arg264′, for ionic interactions with the phosphate groups. These interactions shield almost the entire MTA-CoA molecule from the solvent (Figs. 3A and 4D).

In the second molecule in the asymmetric unit (molecule 4 in Fig. 3B), the electron density for MTA-CoA is somewhat weaker (Fig. 4B), and the phosphopantetheine and MTA groups are not inserted as deeply into the pocket (to be referred to as binding mode B), showing a shift of roughly 1.5 Å in their positions toward the surface of DmdD when compared to binding mode A (Fig. 4C). As a result, two extra water molecules are found at the bottom of the active site pocket for binding mode B (Fig. 4C). In addition, the C-terminal loop and part of helix α11′ of the neighboring monomer are not ordered in the active site for binding mode B (Fig. 2A), which leaves the phosphate groups of CoA exposed to the solvent (Fig. 3B). The adenine base is also partly exposed as Phe255′ in helix α11′ is disordered (Fig. 4C). Moreover, the β9-α5 loop (residues 140–146, including the catalytic Glu141) has conformational differences in the two active sites (Figs. 2A and 4C). These differences are possibly coupled with the shift of the MTA carbonyl group, as Glu141 is positioned directly next to the MTA carbonyl group in both binding modes.

The carbonyl oxygen of MTA is hydrogen-bonded to the main-chain amides of Leu71 and Gly118, the oxyanion hole of this enzyme (Fig. 4C). The aliphatic portion of MTA is located in a mostly hydrophobic pocket, furthest away from the surface of DmdD. The side chains of Leu71, Phe86, Cys90, Trp93, Ile146, and Asn243′ line the surfaces of this pocket. The sulfur atom of the MTA group has polar interactions with the side chain of Asn243′ and van der Waals interactions with the side chains of Phe86 and Cys90. The methyl group on the sulfur atom lies against the Trp93 side chain.

### The binding modes of MMPA-CoA

The position of MMPA-CoA in the first active site (molecule 1) is almost identical to that of MTA-CoA (binding mode A, Fig. 4E). MTA has a double bond between its α and β atoms, and the substituents on this double bond are in the *trans* configuration. MMPA-CoA has a single bond here, but its substituents mostly maintain a *trans* configuration. Therefore, MMPA-CoA is bound to this active site with little conformational disturbances as compared to MTA-CoA.

In the second active site (molecule 4), however, the MMPA group as well as a part of CoA are not inserted into the pocket. Instead, they are on the surface of DmdD and are disordered. This binding mode of MMPA-CoA will not be discussed further here.

### Kinetic studies of DmdD

We next characterized the catalytic activities of wild-type DmdD and the active site mutants (E121A, E141A, and E121A/E141A) toward MTA-CoA, MMPA-CoA and other CoA analogs—acryloyl-CoA, crotonyl-CoA, and pentanoyl-CoA (Fig. 1A). We monitored the hydrolysis of the CoA ester as well as the release of MeSH from MTA-CoA and MMPA-CoA. Wild-type DmdD has strong activity toward MTA-CoA, both for MeSH release (equivalent to hydration of MTA-CoA) and for CoA ester hydrolysis (Table 2). The *k*_cat_/*K*_m_ value for wild-type DmdD is 5×10^6^ M^−1^ s^−1^, ∼10-fold less than that for ECH, which is diffusion limited. This result suggests that DmdD is well adapted for MTA-CoA hydration, which is its physiological activity. In contrast, no MeSH release could be detected with the MMPA-CoA substrate, and CoA ester hydrolysis is also much weaker (∼400-fold lower *k*_cat_/*K*_m_) with this substrate.

**Table 2:** Summary of kinetic parameters.

Reaction	Substrate	Wild-type DmdD	E121A	E141A	E121A/E141A
*K*_m_ (µM)^1^					
CoA ester hydrolysis	MTA-CoA	8.2±2.0	N.A.^2^	N.A.	N.A.
	MMPA-CoA	69.0±16.4	N.A.	N.A.	N.A.
	3-hydroxybutyryl-CoA	119±20			
MeSH release	MTA-CoA	9.4±2.1	N.A.	63.3±12.0	N.A.
	MMPA-CoA	N.A.			
Hydration	Crotonyl-CoA	19.0±3.1			
*k*_cat_ (s^−1^)^1^					
CoA ester hydrolysis	MTA-CoA	44±4	N.A.	N.A.	N.A.
	MMPA-CoA	0.9±0.1	N.A.	N.A.	N.A.
	3-hydroxybutyryl-CoA	13.2±1.6			
MeSH release	MTA-CoA	47±3	N.A.	0.0036±0.0004	N.A.
	MMPA-CoA	N.A.			
Hydration	Crotonyl-CoA	42±3			

1 Values shown are the average and standard deviation from triplicate experiments. Acetyl-CoA, acryloyl-CoA, butyryl-CoA, isobutyryl-CoA, malonyl-CoA and pentanoyl-CoA were also tried as substrates for CoA ester hydrolysis, but no activity was observed. In addition, acryloyl-CoA was not hydrated.

2 N.A. – No activity was detected. The limits for detection were 1.4×10^−4^ s^−1^ and 4.8×10^−5^ s^−1^ for CoA ester hydrolysis and MeSH release, respectively.

The E121A and E121A/E141A mutation abolished MeSH release and CoA ester hydrolysis activities against all the substrates tested (Table 2). Interestingly, the E141A mutant showed very weak (∼88,000-fold lower *k*_cat_/*K*_m_ compared to the wild-type enzyme) but detectable MeSH release activity, although it had no CoA ester hydrolysis activity.

**Figure 5:**
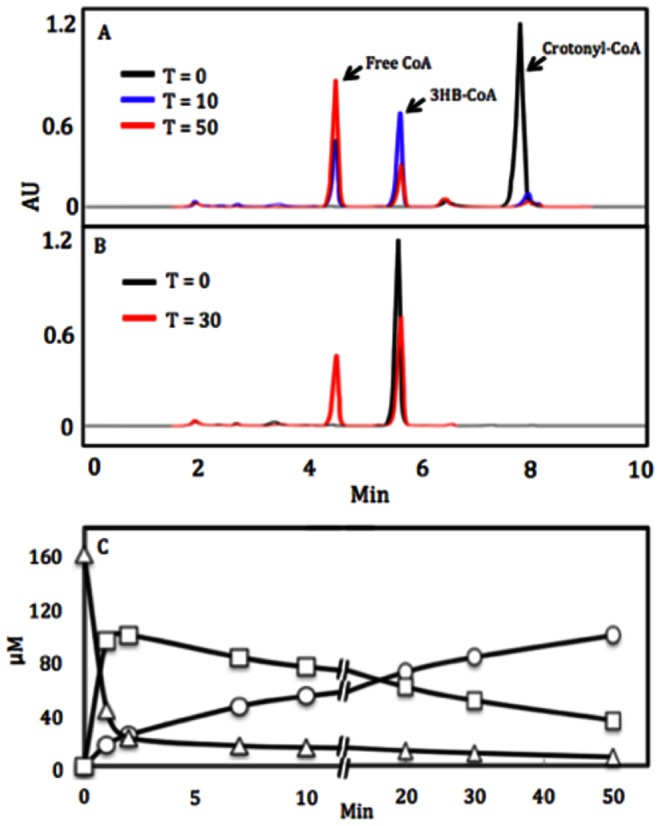
Reactions of DmdD with crotonyl-CoA and 3-hydroxybutyryl-CoA. Chromatography the DmdD reaction products following incubation with 160 µM crotonyl-CoA for 0, 10, and 50 min (**A**) or 3-hydroxybutyryl-CoA (3-HB-CoA) for 0 and 30 min (**B**). AU is absorbance units. (**C**). Reaction time course for the hydration/hydrolysis of crotonyl-CoA by DmdD. Crotonyl-CoA (▵) is consumed at an initial rate of 90 µmol min^−1^ mg^−1^. The formation of 3-hydroxybutyryl-CoA (□) and HS-CoA (○) occurs at initial rates of 72 µmol min^−1^ mg^−1^ and 12 µmol min^−1^ mg^−1^, respectfully. After 2 min the rate of consumption of crotonyl-CoA proceeds at 0.94 µmol min^−1^ mg^−1^. Consumption of 3-hydroxybutyryl-CoA occurs at 2.5 µmol min^−1^ mg^−1^, while formation of free CoA proceeds at 3.3 µmol min^−1^ mg^−1^.

Crotonyl-CoA is an analog of MTA-CoA (Fig. 1A). Wild-type DmdD catalyzed the hydration of this substrate to produce 3-hydroxybutyryl-CoA (Fig. 5), although the *k*_cat_ for this reaction, 42 s^−1^ (Table 2), was much lower than that of the canonical crotonases, which are in the range of 2,000–6,000 s^−1^ [16], [17], [18]. Although the kinetics could not be determined because of competition with the hydration reaction, DmdD also catalyzed the release of CoA from crotonyl-CoA (Fig. 5). Moreover, DmdD catalyzed the hydrolysis of 3-hydroxylbutyryl-CoA with a *k*_cat_ of 13 s^−1^ (Table 2). Because these hydrolysis reactions compete with the hydration of crotonyl-CoA, and it is unclear if they are part of the physiological reactions of this enzyme.

Acryloyl-CoA is one carbon shorter than crotonyl-CoA, but wild-type DmdD showed no detectable activity (hydration or CoA ester hydrolysis) toward this substrate. Pentanoyl-CoA is an isosteric analog of MMPA-CoA, with the replacement of the sulfur atom of MMPA by a methylene group (Fig. 1A). However, wild-type DmdD and the mutants displayed no detectable CoA ester hydrolysis activity toward this substrate. Likewise, we did not observe any hydrolysis activity for wild-type DmdD against acetyl-CoA, malonyl-CoA, butyryl-CoA, and isobutyryl-CoA either.

### Catalytic mechanism of DmdD

**Figure 6:**
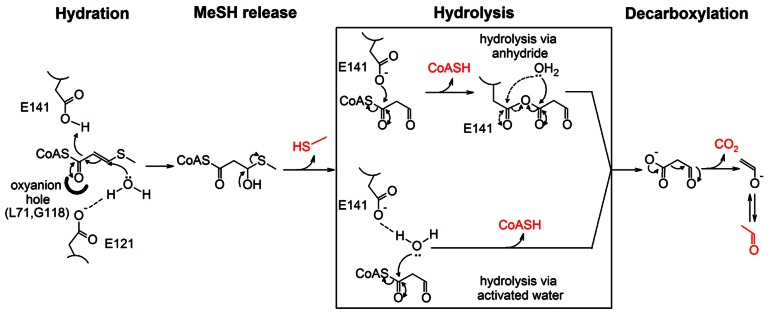
A proposed catalytic mechanism for the hydration and hydrolysis of MTA-CoA by DmdD. The products of the reaction are shown in red. For the hydrolysis of the anhydride in the anhydride mechanism, the hydroxyl group can attack either of the carbonyl carbons, as indicated by the two arrows.

Our studies show that DmdD catalyzes the efficient hydration as well as hydrolysis of MTA-CoA. The hydration reaction is analogous to the canonical crotonase enzymes, and likely employs a similar mechanism, with Glu121 as the general base and Glu141 as the general acid (Fig. 6). The MeSH release activity of DmdD is a spontaneous outcome after the hydration of MTA-CoA, and does not require enzymatic catalysis. This is also supported by the fact that DmdD cannot release MeSH from MMPA-CoA.

Hydroxycinnamoyl-CoA hydratase-lyase (HCHL) is another crotonase that possesses CoA ester hydrolysis activity [7]. The enzyme proceeds through an anhydride mechanism, where an active site carboxylate group attacks the carbonyl carbon of the CoA ester. This produces an enzyme-substrate anhydride, which is then hydrolyzed by a solvent water molecule. Besides the anhydride mechanism, an elimination-addition mechanism has also been observed for the hydrolysis of CoA esters [19]. This involves the abstraction of the α proton of the CoA ester, which gives rise to an enolate. Elimination of CoA produces a ketene intermediate, which is followed by the addition of a hydroxide to generate the acid product. Finally, hydrolysis of the CoA ester by the enzyme CarB most likely proceeds through the attack of the carbonyl carbon by a water molecule activated by an active site glutamate side chain [9].

For DmdD, the Glu141 residue either directly attacks the carbonyl carbon of the CoA ester (the anhydride mechanism) or activates a water molecule for the attack to catalyze CoA ester hydrolysis (Fig. 6). This is supported by our observation that the E141A mutant has weak hydration activity (MeSH release) but no hydrolysis activity (Table 2). At the same time, the strongest hydrolysis activity is observed for MTA-CoA, while MMPA-CoA and crotonyl-CoA are hydrolyzed at much lower rates and pentanoyl-CoA is not hydrolyzed. Therefore, the presence of both the double bond and the sulfur atom in the substrate is important for the hydrolysis activity. MTA-CoA is converted to malonate semialdehyde-CoA after hydration and MeSH release, and the 3-aldehyde group may also be an important factor in stimulating the hydrolysis reaction.

With hydration followed by release of MeSH and hydrolysis to eliminate CoA, MTA-CoA is converted to malonyl semialdehyde by DmdD (Fig. 6). It is expected that this compound can spontaneously decompose, producing CO_2_ and acetaldehyde, thereby explaining the observed products of the reaction.

In summary, we present here the high-resolution crystal structures of DmdD free enzyme and the complex with the substrate MTA-CoA or MMPA-CoA. The structures define the detailed binding modes of the two compounds, providing molecular insights into substrate binding by this enzyme. Our kinetic studies indicate that the unique property of the MTA-CoA substrate is important for the hydration, MeSH release, and CoA ester hydrolysis activities of DmdD. These observations are consistent with the hypothesis that DmdD evolved from an enoyl-CoA hydratase with significant activity for crotonyl-CoA. Acquisition of the MTA-CoA hydration and thioester hydrolysis activities may have resulted in a reduction of the activity toward crotonyl-CoA. Among the MMPA-metabolizing bacteria, only a few possessed homologs with high similarity to DmdD, suggesting that this enzyme may have evolved relatively recently.

## Materials and Methods

### Protein expression and purification

*Ruegeria pomeroyi* DmdD was sub-cloned into the pET26b vector (Novagen) and the recombinant protein, with a C-terminal hexahistidine tag, was over-expressed in *E. coli* BL21 (DE3) Star cells (Novagen), which were induced with 0.4 mM IPTG and allowed to grow at 16°C for 14–18 h. The soluble protein was purified by nickel-agarose affinity and gel-filtration chromatography. The purified protein was concentrated and stored at −80°C in a buffer containing 20 mM Tris (pH 8.5), 250 mM NaCl, 5 mM DTT and 5% (v/v) glycerol. The C-terminal His-tag was not cleaved prior to crystallization.

### Mutagenesis

The E121A and E141A single-site mutants and the E121A/E141A double mutant of DmdD were created with the QuikChange kit (Stratagene) and sequenced to confirm the incorporation of the correct mutation. The mutant proteins were expressed in *E. coli* and purified following the same protocol as for the wild-type protein.

### Protein crystallization

Crystals of wild-type DmdD were grown at 20°C by the hanging-drop vapor diffusion method. The protein solution was at 8.5 mg/ml concentration and was mixed with the reservoir solution with 1∶1 ratio. The reservoir solution contained 100 mM Tris (pH 8.5), 25% (w/v) PEG 3350, and 200 mM NaCl. Fully-grown crystals were obtained three days after set-up. The crystals were cryo-protected in the crystallization solution supplemented with 25% (v/v) ethylene glycol and flash-frozen in liquid nitrogen.

Crystals of the E121A mutant were grown under the same condition as the wild-type protein. The crystals were subsequently soaked for 48 hours in a solution containing 10 mM MTA-CoA or MMPA-CoA, 100 mM Tris (pH 8.5), 35% (w/v) PEG 3350, and 200 mM NaCl, and then flash-frozen in liquid nitrogen. No additional cryo-protectant was used.

### Data collection and processing

X-ray diffraction data on wild-type DmdD free enzyme and the E121A mutant in complex with MMPA-CoA were collected at 100K at the National Synchrotron Light Source (NSLS) beamline X29A on an ADSC Quantum-315r CCD, while the data on the E121A mutant in complex with MTA-CoA were collected on a Pilatus 6M CCD at NSLS beamline X25A. The diffraction images were processed and scaled with the HKL package [20]. All three crystals belong to space group *P*2_1_3, with unit cell parameters of *a* = *b* = *c* = 117.7 Å for the wild-type enzyme. There are two DmdD monomers in the asymmetric unit.

### Structure Determination and Refinement

The structure of wild-type DmdD was solved by the molecular replacement method with the program COMO [21], using the structure of its closest homolog in the PDB, *Rhodopseudomonas palustris* enoyl-CoA hydratase (PDB ID 3HIN), as the search model, with which DmdD shares 45% amino acid sequence identity. The structure refinement was carried out with the program CNS [22], with isotropic atomic temperature factors, and the atomic model was built with Coot [23]. Water molecules were selected automatically by the program. For the free enzyme structure at 1.5 Å resolution, anisotropic atomic temperature factors were then refined, with the program Phenix [24]. The data processing and refinement statistics are summarized in Table 1.

### Sources of CoA thioesters

Butyryl-CoA, isobutyryl-CoA, malonyl-CoA, acetyl-CoA, 3-hydroxybutyryl-CoA and crotonyl-CoA were purchased from Sigma-Aldrich. MTA-CoA and MMPA-CoA were synthesized enzymatically as describe by Reisch *et. al.* [2]. Pentanoyl-CoA was prepared from the acid anhydride using the methods described by Stadtman [25]. Acryloyl-CoA was synthesized from acryloyl chloride (Sigma-Aldrich) as describe by Kuchta & Abeles [26]. CoA thioesters that were synthesized were purified by reverse phase chromatography using an Ultrasphere ODS preparative column (10×250 mm). With the exception of acryloyl-CoA, the column was developed with 50 mM ammonium acetate (pH 6) and a gradient of 2–20% acetonitrile. The CoA thioesters were detected at 254 nm. Fractions containing the CoA thioester were lyophilized, re-suspended in dH_2_O and again lyophilized. For acryloyl-CoA, the column was developed with 50 mM sodium phosphate (pH 7) and a gradient of 2–20% acetonitrile. The fractions containing acryloyl-CoA were then pooled and concentrated 3–4-fold under a stream of N_2_ gas.

### Enzyme assays and kinetic analyses

Enzyme assays were performed in 100 mM HEPES (pH 7.4) with substrate concentrations of 1, 2.5, 5.0, 10, 25, 50, and 150 µM. Reactions were initiated with the addition of enzyme, incubated for 2–10 minutes, quenched by addition of H_3_PO_4_, and briefly centrifuged to remove denatured proteins. Coenzyme-A release and the formation of 3-hydroxybutyryl-CoA from crotonyl-CoA were determined by HPLC using a 4.6×150 mm, 3 µm, Hypersil Gold column (Thermo-Fisher) developed with a linear gradient of 2–20% acetonitrile in 50 mM ammonium acetate (pH 6) over 10 min. A Waters model 2487 UV detector was used at 260 nm. 3-Hydroxybutyryl-CoA was identified by coelution with authentic standard. MeSH release was determined in sealed vials. The headspace was sampled, and MeSH was measured by gas chromatography on an SRI 8610-C gas chromatograph with a Chromosil 330 column (Supelco) with N_2_ carrier gas at a flow rate of 60 ml min^−1^, an oven temperature of 60°C, and a flame photometric detector. A standard curve for MeSH was obtained by suspending sodium methanethiolate (Sigma-Aldrich) in H_2_O. The final values of MeSH produced were then calculated from the sum of the MeSH in the headspace plus the MeSH dissolved in the assay solution. This latter value was calculated from a Henry's constant of 0.1447 at 25°C [27]. In control experiments, the amount of MeSH produced was equal to the amount of HS-CoA detected by HPLC, indicating that MeSH was measured quantitatively.

All enzyme assays were performed with an acid-killed enzyme controls or T0, in which acid was added to the assay buffer prior to the addition of enzyme. Any observed product was then subtracted from the enzymatic rates. However, only very low rates of nonenzymatic hydration of MTA-CoA were observed. Kinetic data were analyzed using SigmaPlot 10.0 with the Enzyme Kinetics module (Systat Software Inc.).
